# Homomorphic inference of deep neural networks for zero-knowledge verification of nuclear warheads

**DOI:** 10.1038/s41598-023-34679-7

**Published:** 2023-05-08

**Authors:** Gabriel V. Turturica, Violeta Iancu

**Affiliations:** Extreme Light Infrastructure-Nuclear Physics, Horia Hulubei National Institute for R&D in Physics and Nuclear Engineering, Bucharest-Magurele, Romania

**Keywords:** Characterization and analytical techniques, Imaging techniques, Information theory and computation

## Abstract

Disarmament treaties have been the driving force towards reducing the large nuclear stockpile assembled during the Cold War. Further efforts are built around verification protocols capable of authenticating nuclear warheads while preventing the disclosure of confidential information. This type of problem falls under the scope of zero-knowledge protocols, which aim at multiple parties agreeing on a statement without conveying any information beyond the statement itself. A protocol capable of achieving all the authentication and security requirements is still not completely formulated. Here we propose a protocol that leverages the isotopic capabilities of NRF measurements and the classification abilities of neural networks. Two key elements guarantee the security of the protocol, the implementation of the template-based approach in the network’s architecture and the use of homomorphic inference. Our results demonstrate the potential of developing zero-knowledge protocols for the verification of nuclear warheads using Siamese networks on encrypted spectral data.

## Introduction

The Cold War’s escalating tensions have seen the world’s superpowers stockpiling over 60,000 nuclear warheads at their peak in 1986^1,2^. Since then, several treaties like the Strategic Arms Reduction Treaty (START) and Strategic Offensive Reduction Treaty (SORT) have reduced this number to a global amount of 13,000, with the USA and Russia holding about 6000 each^3^. Further reduction efforts are built around verification procedures capable of authenticating nuclear warheads while preventing the disclosure of confidential information. Despite decades of research, an optimal verification protocol capable of achieving these goals is still not entirely formulated. The primary constraint related to such protocols stands in the seemingly contradictory formulation of the task: demonstrate to a third party that the item in your possession is authentic without disclosing any information about the item.

In the context of nuclear warhead verification, zero-knowledge protocols aim to prove to an external inspector that the warhead under testing is genuine and has not been tampered with in any way. A hoax warhead can be broadly classified into compositional or geometrical hoaxes. A compositional hoax is a nuclear warhead in which any element was replaced with a surrogate, or the isotopic composition of any component has been altered. On the other hand, in the geometrical hoax case, the elemental/isotopic composition is preserved, but changes to the structural integrity of the warhead have been made. Up to this point, two types of verification protocols capable of identifying hoax warheads have been proposed^4^. The first approach is based on defining a set of absolute attributes as unique identifiers of the warhead. Such attributes contained physical features, like the mass or size of the object, and compositional information, such as isotopic ratios, hidden behind information barriers. The fact that some layer of abstraction contains absolute values that can be correlated to the physical properties of the warhead has left such implementations at risk of tampering. Secondly, given that the attributes are hand-picked, there is the possibility that the selected set of attributes does not uniquely define the nuclear warhead, allowing the chance of fake warheads passing as genuine. The drawbacks of attribute-based methods have forced a shift to template-based verification protocols, which use an already proven sample (template) to validate the warhead under testing. A template-based method implies the ability to quantify the relative difference between two objects on some predefined metrics. Measurements of the appropriate metrics without disclosing any information about the objects ensure zero-knowledge verification.

Several groups have provided template-based protocols using different imaging modalities^4–8^. The proposed Princeton protocol is based on fast neutron radiography to achieve zero-knowledge validation using preloaded superheated bubble detectors^5,6^. Using preloaded detectors ensures the method’s security as the sensitive information is never measured directly. While powerful in its approach, the technique lacks the isotopic specificity to identify compositional hoaxes. An MIT-based group has proposed a different protocol that employs the superior isotopic discrimination capabilities of Nuclear Resonance Fluorescence (NRF) and targets compositional as well as geometrical verification^4^. The method is based on the detection of resonant photons produced in the interaction of an incident beam with a sample material. The photons are generated in specific nuclear transitions that can be uniquely assigned to particular isotopes^9^. The proposed protocol employs a transmission NRF configuration^10^, in which the witness foil characteristics are withheld from the verifier and are used to encrypt the measured spectrum. Unlike the Princeton protocol, the MIT protocol cannot achieve the zero-knowledge standard as the verifier will have access to hashed measurement results.

This work proposes a new template-based protocol built on the isotopic capabilities of NRF measurements coupled with the feature selection and classification abilities of neural networks^11–14^ to achieve zero-knowledge verification. This algorithm combines for the first time machine learning and homomorphic inference to authenticate nuclear warheads. Two elements ensure the security of the protocol, the implementation of the template-based protocol at the architecture level, using Siamese networks, and the use of homomorphic encryption at inference time. The results of this work are presented in two parts: an extensive 2D example demonstrating the capability of Siamese neural networks to address the warhead verification problem and a single-point analysis highlighting the advantages and current limitations of homomorphic inference.

## Results

### Datasets generation

**Figure 1:**
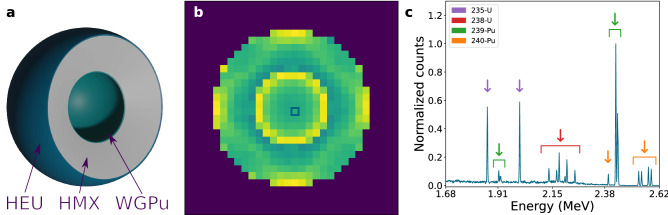
Validation data point. (**a**) Schematic representation of the Black Sea warhead. The warhead is composed of concentric spheres composed of HEU, HMX, and WGPu. (**b**) Monte Carlo simulation results for the Black Sea warhead. The image is obtained by integrating the energy histogram for all eight detectors. (**c**) Normalized energy histogram summed over the eight detectors for the pixel annotated in the middle panel.

Monte Carlo simulations data were used to generate the training, testing, and validation datasets, required to evaluate the capabilities of the proposed verification protocol. The measurement setup was composed of eight detectors uniformly distributed on two rings at 45$^\circ$ and 135$^\circ$ with respect to the incident beam. The sample under testing is positioned in the center of the setup. In this configuration, the detectors will directly record the beam-sample interaction. A continuous beam was approximated as the sum of multiple zero-bandwidth beams tuned on the energies of the selected NRF resonances, to reduce the computational cost. A 19.45 $\times$ 19.45 $\hbox {cm}^2$ measurement scene was raster scanned for a total of 900 points. From the recorded histograms, only the 1.68–2.62 MeV interval was used in the analysis, amounting to 750 bins. As such, a single measurement is defined as a three-dimensional vector with shape [900,750,8]; an input example is shown in Fig. 1. Each measurement vector was normalized in the [0,1] range.

Two distinct datasets were the basis for the training, testing, and validation splits. The training and testing datasets were sampled from a randomly generated dataset composed of 100 scene configurations. The generated scenes contained randomly selected spectral features, like NRF lines, intensities, and branch ratios, as well as geometrical features, like the shape and size of the object. The shape of the objects was sampled out of four possible outcomes: concentric spheres and cylinders, sequential cylinders and rectangular plates (see Fig. S1 in the Supplementary Information). Each shape was composed of three materials, a low Z layer as a surrogate for an explosive placed between two high Z layers as surrogates for U and Pu. Six additional sub-samples were generated from the basic scene configuration, defined as the anchor scene, to account for multiple verification scenarios. Three valid warhead scenes assumed to be affected by experimental uncertainties, compared with the anchor scene, were generated with: increased bandwidth, changes in object position, and under-threshold isotopic changes. The rest of the sub-samples assumed as hoax warheads were: different shapes, different NRF lines, and over-threshold isotopic changes (see Fig. S2 in the Supplementary Information). The isotopic changes were defined with respect to a 10% threshold difference, assumed to be the method’s sensitivity, between the anchor and the generated sub-sample. Based on the architecture of the network, described in details in the following section, each input sample has to be composed of two measurement points with shape [900,750,8] and one true/false label. Six valid/invalid input samples were constructed starting from the anchor and their associated sub-samples. Besides these, two additional samples were built using the increased bandwidth sub-sample and the isotopic changes sub-sample associated with the valid and invalid cases. In total, eight input samples were created starting from each anchor scene. The training/testing datasets were generated using a 0.7 split in favor of the training dataset; the split was made on the anchor level yielding a final shape for the training dataset of [70$\times$8,2,900,750,8].

The validation dataset was composed of nine scenes generated starting from the dimensions and material composition of the Black Sea warhead^15^. The anchor scene was defined as three concentric spheres with the following configuration: weapons grade plutonium (WGPu; 5% $^{240}$Pu), explosive (HMX), highly-enriched uranium (HEU; 5% $^{238}$U). Out of the nine scenes, two were anchor scenes, identical scenes repeated to account for the simulation uncertainties. Three isotopic hoax scenes were generated by replacing the anchor’s WGPu (5% $^{240}$Pu) with: fuel grade plutonium (14% $^{240}$Pu), reactor grade plutonium (25% $^{240}$Pu), and HEU. Four geometric hoax scenes were created by altering the anchor geometrical configuration: removing one and two-pixel thickness from the low Z sphere and changing the object’s shape from a sphere to sequential cylinders and rectangular plates. For each of the nine samples, two valid sub-samples were produced with: increased bandwidth and changes in object position. A validation dataset with shape [32,2,900,750,8] was constructed based on the simulated data.

### Neural network architecture

**Figure 2:**
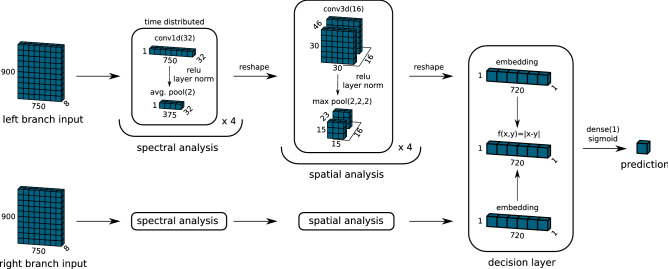
Siamese network architecture for the 2D analysis. The left and right branches have the same structure with shared weights and biases. The figure highlights the three components of the network: spectral analysis, spatial analysis, and the decision layer. The activation shapes shown for the spectral and spatial analysis correspond to the first block.

A Siamese network was selected to implement the template-based protocol at the architecture level. The schematic depiction of the selected model is shown in Fig. 2. The Siamese network is composed of two identical branches with shared weights that take as input the measurements of the template and the object under testing and outputs a one-dimensional feature vector. The feature vector is then passed to a common decision layer to extract a similarity score. The main advantage of this architecture stands in the fact that the prediction is based on the similarity of the extracted features instead of a direct classification. This ensures that the model’s capacity is not used to memorize specific features learned during training, like the positions or relative intensities of particular peaks.

The model is divided into three parts based on their objective: the spectral and spatial analysis parts, and the decision layer. The first part extracts the spectral features from each one of the 900 measurement samples of the input. This is accomplished using a TimeDistributed block that applies the same Conv1D filter to each sample, followed by ReLU, LayerNormalization, and AveragePooling1D. The spectral part is composed of four such blocks that transform the input from [900,750,8] to [900,46,32]. The output of the spectral part is reshaped to [30,30,46,32] and passed on for spatial analysis. The spatial analysis part captures correlations between the individual pixels to extract information about the shape and position of the object. This is done using four convolutional blocks, each composed of Conv3D layers followed by ReLU, LayerNormalization, and MaxPooling3D. An exception is made for the last block that skips the pooling layer. The output of the spatial analysis is flattened to a one-dimensional feature vector with a length of 720 elements. The decision layer calculates the absolute difference between the two feature vectors passed on from the two branches. A single unit Dense layer followed by a Sigmoid activation squashes the vector to a single-valued similarity prediction in the [0,1] range.

### Homomorphic encryption

Homomorphic encryption algorithms are constructed around noisy ciphertexts that support performing mathematical operations without the loss of encryption. The ability to perform encrypted computations guarantees the preservation of data privacy for each step in the processing pipeline. The main limitation associated with homomorphic encryption was the increasing amount of noise with each operation; any additional step above the maximum noise level renders the ciphertext undecryptable. These schemes, known as somewhat homomorphic schemes, would allow only a finite number of homomorphic operations. The first implementation of a fully homomorphic encryption scheme is described by the seminal work of Gentry^16^. Gentry’s contribution was the introduction of bootstrapping; this technique allowed controlling the noise associated with each successive homomorphic operation. Following Gentry’s result, multiple schemes were developed^17^, among them the Fast Fully Homomorphic Encryption Over the Torus (TFHE)^18^. The Concrete library^19^, a TFHE-based scheme that implements the concept of programmable bootstrap was used throughout this work. The programmable bootstrapping is a key concept that allows fine control of the associated noise and enables the possibility to evaluate any arbitrary function homomorphically.

In its current implementation, the Concrete library contains most of the elements required to implement neural network architectures. In terms of mathematical operations, the library allows addition and subtraction between ciphertexts, addition and multiplication between ciphertext and plaintext, and evaluation of an arbitrary function on a ciphertext through programmable bootstrapping. A homomorphic inference scheme can be implemented using these capabilities in which the weights and biases are plaintexts, while the input, output, and all the intermediate values are encrypted. Two main limitations currently restrict the use of homomorphic inference on complex network architectures: the computational overhead and the 7-bit computational threshold. The computational overhead does not pose a significant issue for the current work, as real-time inference is not necessarily required for the protocol. The 7-bit limit imposes significant constraints on the maximum network accumulator values, restricting the size of the network.

In order to demonstrate the potential of using homomorphic inference for the warhead verification problem while satisfying the 7-bit limitation of the library, a scaled-down version of the network shown in Fig. 2 was implemented (see Fig. S3 in the Supplementary Information). This network targets only the spectral part of the analysis and uses a [1,750,1] vector as input, extracted as a single pixel and a single detector channel out of the complete [900,750,8] sample. The selected network is composed of five blocks, using sequential layers of Conv1D, ReLU1, and AveragePooling1D. All Conv1D layers employed ten convolutional filters, using a kernel size of 3. ReLU1 was selected to restrict the maximum values of the activations flowing through the network. The pooling layer used a pool size of 4 for all but the last block, which used a value of 2. The flattened results of the two branches, features vectors with size 10, are passed on to the decision layer. A single unit Dense layer followed by a Sigmoid activation squashes the vector to a single-valued encrypted prediction in the [0,1] range.

The network was trained in plain using samples from all 100 training/testing with a quantization level of 5 bits (see Fig. S4 in the Supplementary Information). Out of the available sub-samples, the training was made using two samples as valid, re-sampled anchor and under-threshold isotopic changes, and two samples as invalid, different NRF lines and over-threshold isotopic changes. The validation dataset was composed of 34 samples, generated from the Black Sea model. The samples were obtained by applying isotopic variations to the materials described in the datasets generation section.

### Measurement protocol

Constructing realistic protocols around the described methodology does require extensive details about dismantlement treaties and implementation requirements, which fall outside the scope of this work. The following description represents a simplistic view of such a procedure. The proposed protocol involves three entities participating in the verification procedure: host, inspector, and an overseeing authority. The host is the owner of the warhead under testing and of the measurement setup. It is assumed that the measurement will be made in the host’s facility. The inspector is the entity that aims to verify the authenticity of the sample in the possession of the host. The inspector will arrive at the host’s facility accompanied by a genuine warhead authenticated by other means^4^. The overseeing authority is an independent organization that supervises the verification procedure and is the verification algorithm’s owner. The overseeing authority allows the involved parties to perform computations using plain and encrypted data. The host and the inspector can submit a number of test measurements, which are required to validate the measurements station and the validation algorithm. Limited access to the verification algorithm restricts the possibility of the host or the inspector to discover and exploit any shortcomings of the validation algorithm. After both parties agree on the validity of the setup and analysis algorithm, the warhead under testing is measured. Every exchange containing sensitive data between the parties and the overseeing authority is made using encrypted data. As such, the data that the overseeing authority has access to are encrypted measurement results and the number of measurements done. Access to confidential information is impossible as long as the 128-bit encryption is not broken. Furthermore, TFHE encryption schemes are based on learning with errors (LWE) and ring-LWE, which are naturally quantum-resistant^20^.

## Discussion

In the context of nuclear spectroscopy, several factors can interfere with a measurement’s integrity. A neural network that can be used for an automatic verification procedure should be able to account for experimental edge cases and correctly assess the validity of the sample. In a zero-knowledge protocol scenario, the problem is amplified as histograms cannot be visualized in plain to identify common experimental problems, like poor resolution due to noise or improper sample positioning. The neural networks selected in this work are capable of dealing with these issues, as it is detailed in the following sections.

**Figure 3:**
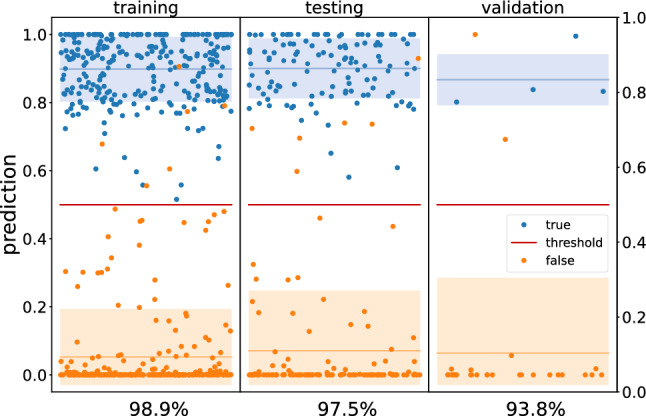
Prediction results for the training, testing, and validation datasets for the 2D analysis. The true and false labels are associated with blue and orange, respectively. The colored regions highlight the associated mean and standard deviation. The x-axis label indicates the prediction accuracy. The data points are randomly distributed along the x-axis for visualization purposes.

The proposed verification protocol uses input data from 2D scenes obtained in a single projection measurement. However, to satisfy the homomorphic library’s 7-bit limitation, a downscaled version of our network was used for the single-point analysis. Hence the discussion is focused on the performance evaluation for the 2D analysis using plain inference and for the single-point analysis using both plain and encrypted data. The results of the inference procedure for the 2D scene are shown in Fig. 3. The figure contains the training, testing, and validation results divided by labels. The network can achieve a 93.8% total accuracy on the validation dataset (see Fig. S5 in the Supplementary Information for additional performance metrics). One thing to note is that the network was trained under a binary weight constraint that limited the network’s performance. Preliminary results, not covered in this work, using 32-bit float weights with similar architecture showed improved results.

In terms of the overall accuracy, the verification protocol can be significantly enhanced if multiple projections are involved. The generality of the Siamese architecture enables the possibility of analyzing measurement samples for an arbitrary number of projections. This might be required as protection against geometric hoaxes that could mimic the template along a predefined projection^4^. The capability of a neural network to analyze high dimensional data also enables the possibility to use tomographic data as input that can yield additional improvements in accuracy.

**Figure 4:**
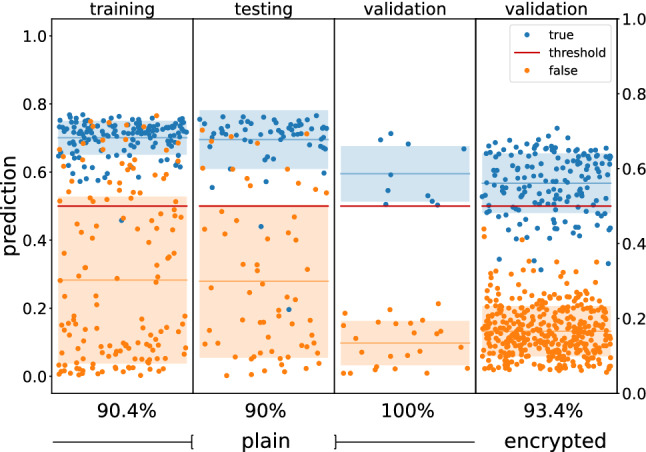
Prediction results for the training, testing, and validation datasets for the single-point analysis. The first three panels contain predictions obtained using plain inference, with the fourth one containing results from encrypted inference. The true and false labels are associated with blue and orange, respectively. The colored regions highlight the associated mean and standard deviation. The x-axis label indicates the prediction accuracy. The data points are randomly distributed along the x-axis for visualization purposes.

The outcome of the inference procedure using plain and encrypted data for single-point analysis is summarized in Fig. 4. Plain inference results for the training and testing datasets show accuracies around 90%. The slight drop in accuracy can be associated with two factors: the limit on the capacity of the network and the loss of information due to the quantization of the input. Besides the accuracy, the limited capacity of the network can be observed in the inability to output values close to 0 and 1, with mean errors around 0.2 and 0.7, respectively. The computational overhead related to the encrypted inference restricted the procedure only for the validation dataset. The encrypted validation results were obtained by running the inference procedure 16 times using the same validation dataset. This step was required to account for the evaluation uncertainties associated with the noise of the homomorphic scheme (see Figs. S6 and S7 in the Supplementary Information). The obtained results for the validation dataset using plain and encrypted data are 100% and 93.4%, respectively.

**Figure 5:**
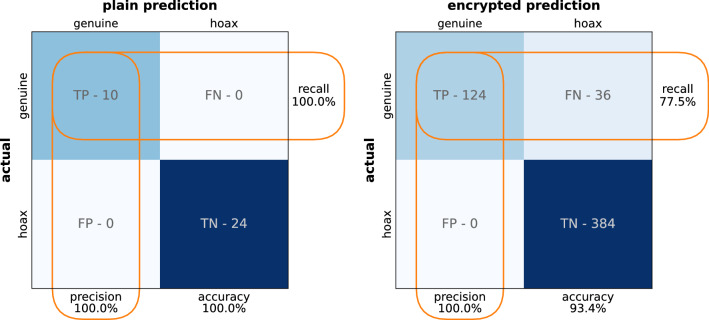
Performance metrics for the single-point analysis of the validation dataset using plain and encrypted inference.

The performance metrics for the single-point analysis using plain and encrypted inference on the validation dataset are shown in Fig. 5. The 100% accuracy obtained for the plain inference translates to the same value for precision and recall. On the encrypted inference, the 93.4% accuracy translates to 100% precision and 77.5% recall. An increase in recall is expected if the 7-bit limitation is exceeded, and the inference will be made using full-precision neural networks. In the context of warhead validation, the high precision highlights the ability of the model to identify and reject hoax objects. On the other hand, the recall value points to the fact that the model will misclassify valid warheads. While not ideal, the misclassification of a warhead would raise alarms that will prompt more thorough searches that would lead to the correct warhead classification. It is important to note that most analysis methods will not be able to achieve perfect performance metrics in realistic scenarios. Hence, complementary methods that enhance the verification performance should be in place to provide the required redundancy.

Despite the reduced complexity of the input, the single-point neural network underperforms in comparison to the 2D neural network on the training and testing dataset. This is directly correlated to the 7-bit limitation of the homomorphic library. An additional increase in the bit rate will alleviate these problems and will allow the possibility of performing homomorphic inference using the full-sized network. The current 7-bit limitation its imposed by the computational load of the bootstrapping operation, which grows roughly exponentially with the length of the plain message. Solutions for speeding up FHE schemes are proposed on the software level, like parallel arithmetic^21^, and at the hardware level with optical computing. Specialized hardware would enable inference on complex full precision networks as several orders of magnitude improvements in processing times are expected^19^.

The results of our work demonstrate the potential of using Siamese neural networks for zero-knowledge verification of nuclear warheads. Developing simulation-based models with excellent performance is a key building block for constructing models based on experimental data. An ideal training scenario for a proof of concept implementation would make use of realistic Monte Carlo simulations for generating pre-training data, followed by training on experimental measurements^12^. Moreover, the generality of our approach enables the ability to further extend the model with additional modalities based on spectral comparison, like neutron-induced nuclear resonance measurements^8^.

The second key feature that guarantees the security of the proposed protocol is the use of homomorphic inference. The ability to perform mathematical operations on encrypted data enables the possibility to apply complex algorithms while preserving data privacy. Our work demonstrates the possibility of achieving zero-knowledge homomorphic inference on spectral data using a complex Siamese architecture. Good accuracy results were obtained for the single-point analysis, using a limited implementation of our model, despite the 7-bit limitation currently imposed by the library. Solutions for extending the current limit are already in the works and will provide the ability to perform homomorphic inference of highly complex architectures.

## Methods

### Datasets generation

The datasets used for the training, testing, and validation of the proposed models were generated using Geant4 version 10.06^22^. G4Penelope classes were used to model the low-energy electromagnetic interactions. The NRF interaction was simulated using the experimentally validated implementation described by Negm^23,24^. The incident beam was modeled as an approximation for a continuous spectrum that can be experimentally obtained using bremsstrahlung or laser Compton scattering. The detectors, part of the measurements setup, were modeled after a 150% relative efficiency high-purity germanium detector. Each of the 900 pixels of a sample was simulated using $4\times 10^7$ incident photons, evenly distributed between the selected NRF lines. Six additional sub-samples were generated starting from the anchor scenes; all were simulated, but the increased bandwidth sub-sample that was obtained by re-sampling the anchor under a Gaussian function. The computations were performed using 300 CPU threads for an approximate total time of 30 days.

### Neural networks

The neural network architectures were implemented using the Keras framework^25^. The training was done using Adam, with a $3\times 10^{-4}$ learning rate, under a contrastive loss function. Due to limitations attributed to the homomorphic inference part the network weights were binarized using the open-source package Larq^26^. The weight values were restricted to $-1$ and 1 using the SteSign quantizer, the biases of the networks were left unquantized. The best model was selected based on the minimal loss on the test dataset from 2000 epochs of training. The computations were performed on a system with two NVIDIA GeForce RTX 2080 Ti GPUs.

### Homomorphic inference

The training procedure for the single-point neural network was made using the procedure described above. Following the training, the single-point neural network architecture was implemented in Rust^27^ for plain and encrypted inference. All the array operations required for the inference were carried out using the ndarray library 0.15.4. The encrypted inference process was implemented using the FHE library Concrete version 0.1.11. The encoder ranges required for the encrypted inference were obtained based on the minimum/maximum values observed during the plain inference on the training and testing datasets. The selected encryption parameters offer a 128-bit security level. The validation dataset for the plain inference was composed of 34 samples. The validation for the encrypted inference was carried out 16 times, using the complete dataset in order to account for the evaluation uncertainties. The encrypted inference compute time required to run the validation dataset was about 350 h on a single CPU thread.

### Threat model

The assumptions of the proposed protocol lead to the following threat model. First, the exchange of sensitive information between the involved parties and the overseeing authority is made using encrypted data, which preserves data privacy. A semi-honest overseeing authority could extract information from the plain-text model, related to the training data and model stability but cannot generate adversarial samples. Second, we assume that the protocol steps from the experimental measurement to the analysis algorithm are not compromised. In that case, the attack path for a malicious host requires the generation of a hoax object. In a black-box scenario where the host does not have direct access to the network, generating adversarial samples would require an abundant number of queries. Such attacks cannot be considered a vulnerability as the number of queries could be easily limited at the protocol level. The system is susceptible to gradient-based attacks in a white-box scenario in which the host gains direct access to the network. Under these conditions, the host can use the genuine warhead to generate adversarial spectra that could deceive the classifier. However, given the statistical nature of spectroscopic measurements and the specificity of nuclear resonances, most of the generated adversarial spectra will not describe realistic objects and cannot be used to reconstruct hoax objects. Currently, the proposed protocol describes the verification process at a conceptual level. The threat model outlines only a limited view of the challenges associated with the verification of nuclear warheads. Several key elements, such as physical security and encryption key handling, fall outside the scope of this paper. These implementation details are essential to ensure the protocol’s security and will be considered in future studies.

The use of neural networks in security-critical environments raises additional challenges as the lack of interpretability and model stability. The robustness of the network is especially important in the context of our work, as small perturbations associated with the homomorphic inference can lead to incorrect predictions^28^. Robust training techniques and ensemble learning have been proposed to generate models with improved stability, and state-of-the-art performance^28–30^. Additionally, network instabilities can be further reduced by using multiple projections and imaging modalities that will significantly decrease the uncertainties associated with the inference of a single measurement. Further studies are required to assess and enhance the stability of homomorphic inference for Siamese networks in the context of zero-knowledge verification of nuclear warheads using spectroscopic data.

## Supplementary Information

Supplementary Information.

## Data Availability

The datasets used and/or analysed during the current study are available from the corresponding author on reasonable request.
