# Supplementary material for: Homomorphic inference of deep neural networks for zero-knowledge verification of nuclear warheads
Source: Sci Rep. 2023 May 8;13:7464. doi: 10.1038/s41598-023-34679-7 (PMC10167340; doi:10.1038/s41598-023-34679-7)
Supplement: Supplementary file 1 — Supplementary Information. [file 41598_2023_34679_MOESM1_ESM.pdf]

Supplementary information

Homomorphic inference of deep neural networks for zero-knowledge verification of nuclear warheads

Gabriel V. Turturica and Violeta Iancu

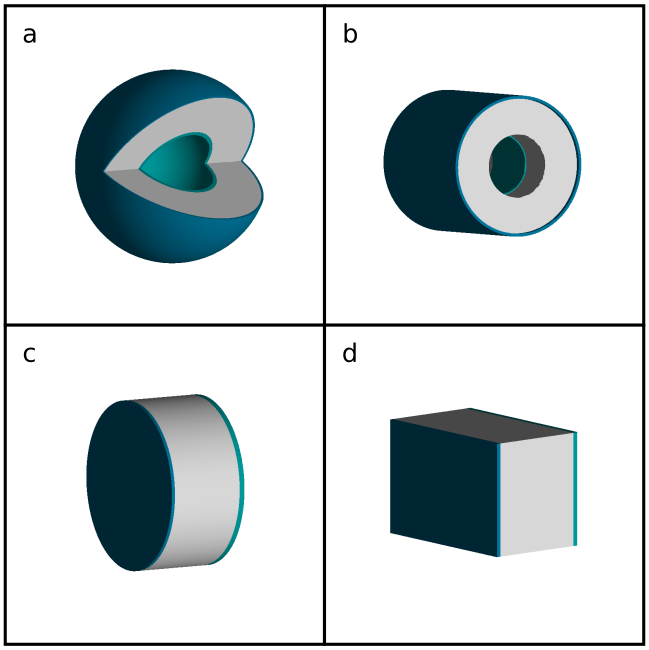

Figure S1: The four possible geometries selected as potential warhead shapes for the generation of the training and testing datasets. Each shape was composed of three materials, a thick low Z layer (gray) as a surrogate for an explosive placed between two thin high Z layers (blue and green), as surrogates for U and Pu. The geometrical dimensions of each layer were randomly selected. Dimensional constraints were applied to satisfy the layers configuration and the scene size of 19.45×19.45 cm^2.

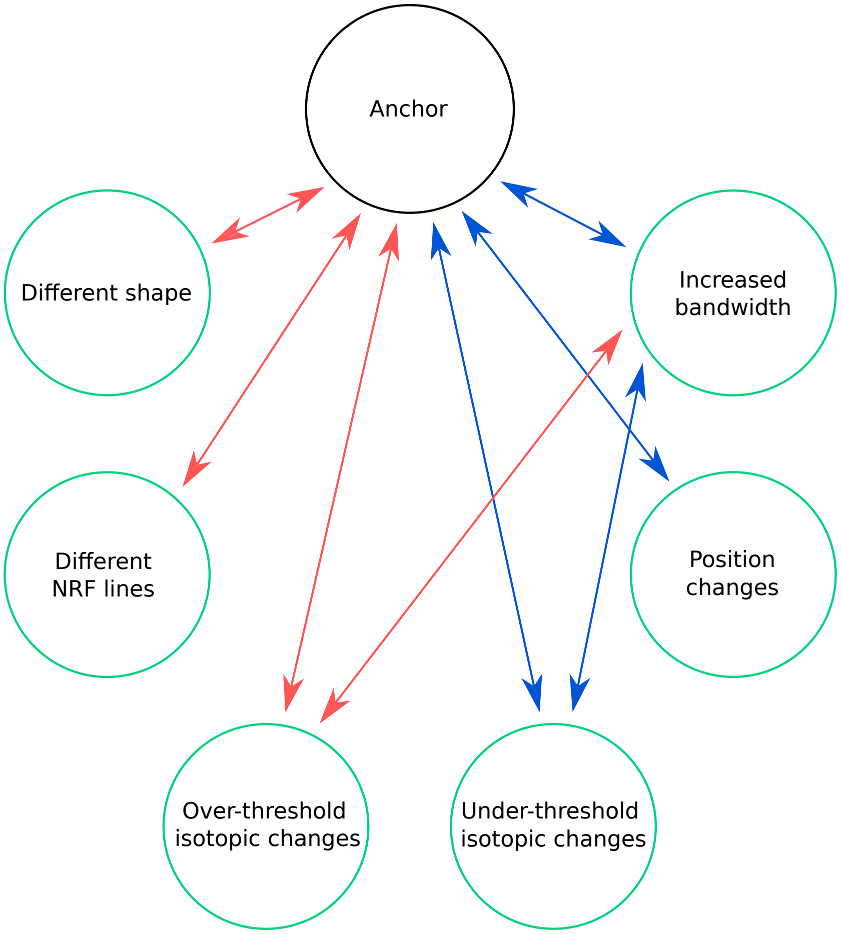

Figure S2: Input sample generation for training and testing. The anchor (black circle) stands as the basic scene configuration. The sub-samples (green circle) were generated by modifying the anchor and are used to generate the input samples. Each individual input sample is composed of measurement data from two scenes and a true and false label. Eight input samples, denoted by the arrows, were built from the available configurations. The red and blue arrows highlight the true and false input samples, respectively.

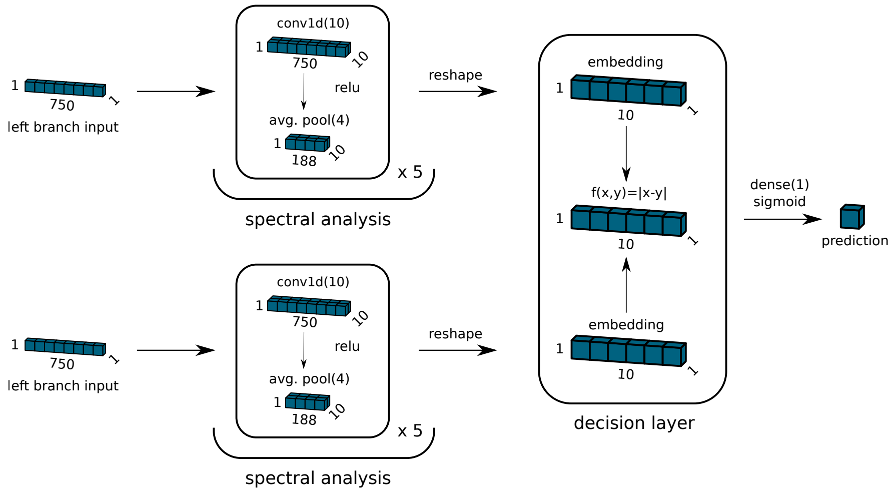

Figure S3: Siamese network architecture for the single-point analysis. The left and right branches have the same structure with shared weights and biases. The figure highlights the two components of the network: the spectral analysis and the decision layer. The activation shapes shown for the spectral analysis correspond to the first block.

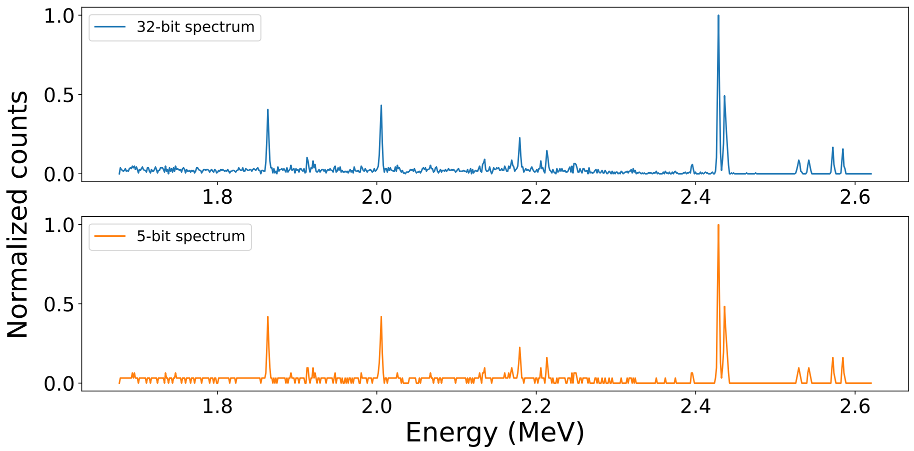

Figure S4: Single-point analysis input examples, histograms in full-precision (top) and 5-bit precision (bottom).

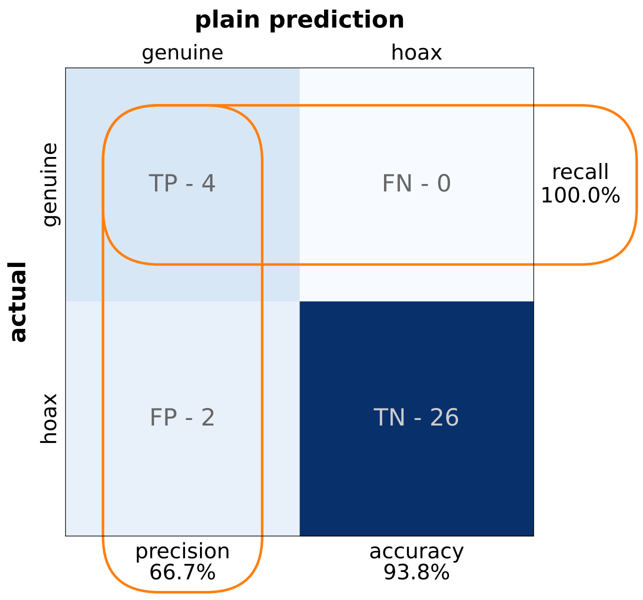

Figure S5: Performance metrics for the 2D analysis of the validation dataset using plain inference.

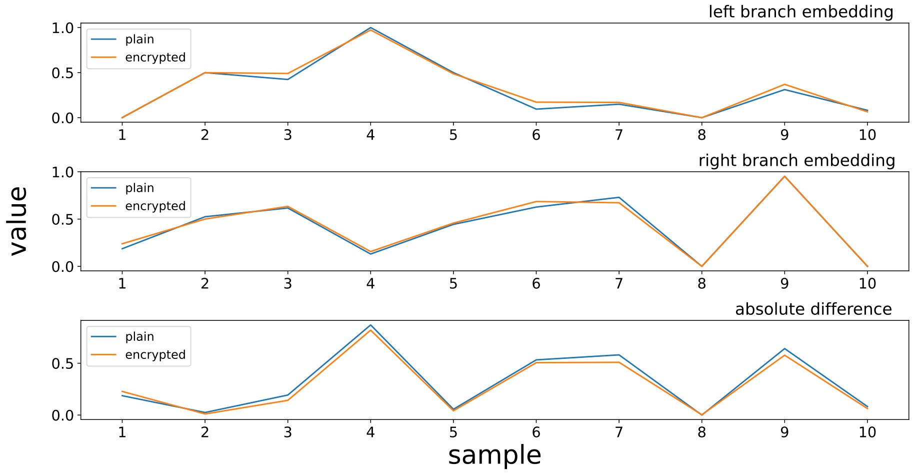

Figure S6: Embedding results for plain and encrypted inference using a single-point input sample. The figure highlights the differences in the evaluated embeddings associated with the noise of the homomorphic scheme. For this sample, the encrypted inference achieved the result expected from the plain inference.

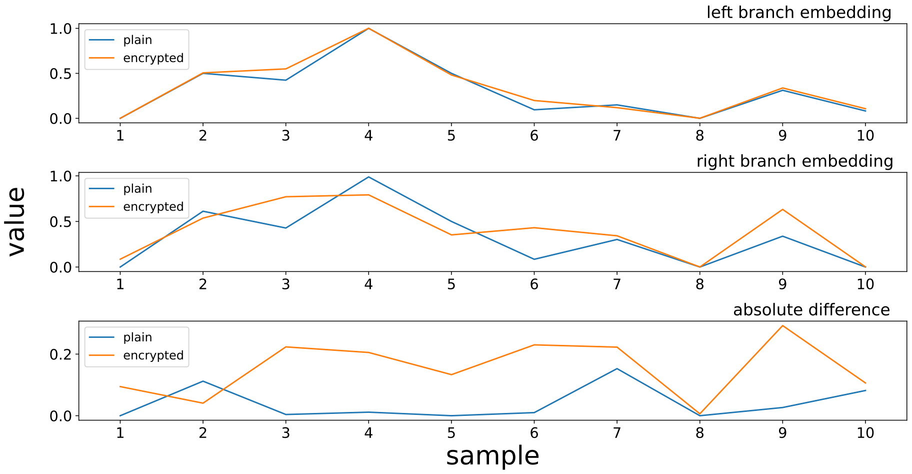

Figure S7: Embedding results for plain and encrypted inference using a single-point input sample. The figure highlights the differences in the evaluated embeddings associated with the noise of the homomorphic scheme. For this sample, the encrypted inference failed to achieve the result expected from the plain inference.
